# Management factors affecting physical health and welfare of tourist camp elephants in Thailand

**DOI:** 10.7717/peerj.6756

**Published:** 2019-04-25

**Authors:** Pakkanut Bansiddhi, Korakot Nganvongpanit, Janine L. Brown, Veerasak Punyapornwithaya, Pornsawan Pongsopawijit, Chatchote Thitaram

**Affiliations:** 1 Center of Elephant and Wildlife Research, Chiang Mai University, Chiang Mai, Thailand; 2 Department of Veterinary Biosciences and Public Health, Faculty of Veterinary Medicine, Chiang Mai University, Chiang Mai, Thailand; 3 Center for Species Survival, Smithsonian Conservation Biology Institute, Front Royal, VA, USA; 4 Department of Food Animal Clinic, Faculty of Veterinary Medicine, Chiang Mai University, Chiang Mai, Thailand; 5 Excellent Center of Veterinary Public Health, Chiang Mai University, Chiang Mai, Thailand; 6 Department of Companion Animal and Wildlife Clinics, Faculty of Veterinary Medicine, Chiang Mai University, Chiang Mai, Thailand

**Keywords:** Health, Welfare, Management, Asian elephant, Thailand, Elephant camp

## Abstract

**Background:**

Variation in management across elephant camps likely has differential effects on the well-being of elephants.

**Methods:**

This study calculated body condition, foot health and skin wound scores (WSs) for 122 elephants from 15 elephant camps in Chiang Mai province, and examined relationships to management factors using a multi-variable modeling approach.

**Results:**

The majority of elephants had high body condition scores (BCS) indicative of being overweight or obese, mild foot problems, but few visible wounds. Females had higher BCSs than males, as did elephants provided a water source at night. Increasing age was associated with higher foot and WSs. Higher WSs were observed in about a quarter of the cases where mahouts carried a hook. Wounds related to saddle riding were rare. Elephants that rested on sand floors at night had a decreased risk of high WSs compared to elephants that rested on compact dirt floors.

**Discussion:**

Findings emphasize the need for elephant camps to adjust management activities that negatively affect body condition (e.g., feeding too many sweet treats), foot health (e.g., hard substrates) and wounding (e.g., misuse of equipment) to improve health and welfare of this population.

## Introduction

The elephant is important to Thai society and has been a national icon for centuries. Initially used for teak harvesting following a 1989 logging ban, elephants were brought in to permanent camps that catered to tourists by providing rides, animal encounters and entertainment through shows (Bansiddhi et al., 2019). Today, there are 3,783 captive elephants in Thailand with almost 95% of them privately owned (Asian Elephant Specialist Group, 2017). According to data sources from the National Institution of Elephant Research and Health Service, in 2017 there were 2,673 elephants working in 223 tourism venues throughout the country. Chiang Mai is the largest city in the north and has the highest numbers, with 892 elephants in 82 venues (Bansiddhi, 2019).

The welfare of captive elephants has been a recent topic of debate among animal managers, conservationists, scientists, the general public, animal welfare/rights groups and the media. Common concerns centering on the welfare of elephants in tourism in particular are complex in their nature and impact, and call for urgent scientific evaluation to identify realistic solutions to ensure the sustainable and ethical management of captive elephants in the future (Asian Captive Elephant Working Group, 2017). In Thailand, the conditions for working elephants and their access to accommodation, appropriate food and veterinary care are highly variable (Duffy & Moore, 2011), with no enforceable standards. Our survey of elephant camps in northern Thailand revealed considerable variation in management practices; that is, work activities (e.g., feeding, bathing, walking, riding and shows) and hours, housing and rest areas, floor types during the day and night, chaining restraint, use of hooks, diets and nutrition, feeding regimens and watering (Bansiddhi et al., 2018). Such variation likely has differential effects on the well-being of these elephants, which can be assessed through evaluations of welfare indicators like body condition, foot health and wounding.

In recent years, studies of management factors related to the health and welfare of elephants have been conducted in western zoo settings, identifying obesity, poor foot health, ovarian acyclicity and stereotypies as significant problems (Carlstead et al., 2013; Harris, Sherwin & Harris, 2008). A recent epidemiological approach in the U.S. evaluated husbandry and management factors impacting animal-based indicators; that is, physical (body condition, foot and musculoskeletal health), behavioral (stereotypies, walking, recumbence) and physiological (ovarian cycling, prolactin, cortisol) outcomes. Results suggest that, for zoo elephants, good welfare is supported by exercise and walking opportunities, unpredictable feeding schedules, natural substrates, large and compatible social groups, high diversity in feeding and enrichment, large and complex enclosures, and positive elephant-keeper relationships (Greco et al., 2017; Haspeslagh et al., 2013; Meehan et al., 2016; Morfeld & Brown, 2017).

A few studies have examined factors affecting the welfare of tourist elephants in Asia, but none comparable to the large-scale zoo studies. In India, Varadharajan, Krishnamoorthy & Nagarajan (2016) examined husbandry practices and daily routines, including the type of activity (e.g., ceremonial rituals), conspecific socialization, duration of chaining, resting, feeding, bathing, walking and drinking. They found that stereotypic behavior (i.e., weaving, head bobbing and pacing) significantly increased with daily rituals in temples, resting and to some extent feeding. It was suggested that elephants become frustrated with performing daily rituals and when chained during non-working time. In Thailand, a study conducted two decades ago by Chatkupt, Sollod & Sarobol (1999) assessed body condition of elephants in Chiang Mai, Phuket, Bangkok and Ayutthaya. Body condition was better in elephants from Chiang Mai where elephants usually received adequate shade, were housed on softer surfaces, and tended to work fewer hours. Common health problems associated with working conditions in Thailand are wounds, particularly those associated with riding and restraint equipment. Magda et al. (2015) found the prevalence of cutaneous lesions in anatomical regions in contact with saddle-related equipment (i.e., neck, girth, back, tail) was related to the use of rice sacks as padding material, longer working days and the provision of a break (possibly because elephants with active lesions were rested more often). Another effect of work and husbandry practices on captive elephants is foot problems (Angkawanish et al., 2009; Lahiri-Choudhury, 2008). Chronically wet or dirty conditions and steep inclines on trekking trails present physical hazards that can damage the elephants’ feet (Chatkupt, Sollod & Sarobol, 1999).

Given the need for more comprehensive, objective, science-based evaluations of elephant welfare in tourism, the objectives of this study were to: (1) assess body condition, foot health and the prevalence of skin wounds of elephants used in tourism in Northern Thailand; and (2) determine how these health parameters are related to tourist camp management factors using a multi-variable modeling approach.

## Materials and Methods

### Study animals and work activities

This study was approved by the Institutional Animal Care and Use Committee, Faculty of Veterinary Medicine, Chiang Mai University, Chiang Mai, Thailand (license number; S43/2559). All experiments were performed in accordance with relevant guidelines and regulations. Data were obtained on 122 healthy elephants (33 males and 89 females) from 15 elephant camps in Chiang Mai (Table 1). The age of elephants ranged from 5 to 65 years. There were five types of work or tourist activities that elephants were involved in (Table 1): riding with a saddle, riding bareback, no riding but some tourist interactions, observation only, and elephant shows as described by Bansiddhi et al. (2018). There were five elephants from two camps that did not work or interact with tourists at all. One was a bull used only for breeding that was tethered most of the time in a shed structure and walked ∼1 km for exercise and to forage for 25 min/day. Four elephants were too aggressive to engage in any camp activities. They were kept in a nearby camp under the control of their mahouts during the day, in enclosures at night, and were walked for 20–30 min/day (∼2 km).

**Table 1:** Summary of elephants in 15 elephant camps in Chiang Mai province.

Camp No.	Number of total elephants	Number of participating elephants			Number of elephants in each type of work					
		Male	Female	Total	Riding with a saddle	Riding bareback	No riding	Observation	Show	No work
1	4	0	3	3			3			
2	5	2	3	5		5				
3	5	0	4	4			4			
4	6	0	5	5	5					
5	6	2	3	5	1	3			1	
6	7	1	4	5			5			
7	9	0	5	5		4	1			
8	10	1	5	6		3	3			
9	15	1	5	6		6				
10	35	3	9	12		12				
11	46	5	9	14		13				1
12	52	6	6	12	10				2	
13	65	0	15	15			4	7		4
14	66	6	6	12	12					
15	76	6	7	13	12				1	
Total		33	89	122	40	46	20	7	4	5

### Questionnaire interviews

Elephant camps were a subset of those in a previous study (Bansiddhi et al., 2018). Questionnaire interviews with camp owners, managers, and/or camp veterinarians were performed to record information about camp activities, location, programs for tourists, numbers of elephants and elephant management (e.g., nutrition, feeding, water, rest area, working and health care) (S1). Questionnaire interviews with mahouts gathered information on management of their specific elephants: work routine, restraint, rest area, feeding, watering and health care (S2). Interviewers and observers were veterinarians experienced in working with captive elephants from the Faculty of Veterinary Medicine, Chiang Mai University.

### Physical health and welfare parameters

Elephants were examined every 2–3 months for a total of six times between February 2016 and May 2017, and given scores for body condition, foot health and skin wounds. One author (PB) performed all veterinary examinations. At the time of each examination, current management and work activity information was recorded for each elephant.

#### Body condition score

A 5-point scale developed by Morfeld et al. (2016) was used to assess a body condition, with 1 representing the lowest and 5 representing the highest levels of body fat.

#### Foot health score

Foot health was scored using a scale adapted from Harris, Sherwin & Harris (2008) and the British and Irish Association of Zoos and Aquariums Elephant Welfare Group described by Todd (2015) (Table 2). Each foot was given a score of 0 (no problem), 1 (mild problems), 2 (moderate problems) or 3 (severe problems). The overall score of each elephant was the highest score from all four feet as described by Todd (2015). The number, location and direction of nail cracks were noted.

**Table 2:** Scoring system for assessing foot health adapted from Harris, Sherwin & Harris (2008) and the British and Irish Association of Zoos and Aquariums (BIAZA) Elephant Welfare Group as described by Todd (2015).

Score	Description
0 (normal)	No lesions, normal nails
1 (mild)	Uncomplicated nail cracks (small cracks which did not extend into the cuticle), mild overgrowth of nails or cuticles, mild dry cuticles, mild disfigured nails or mild injuries
2 (moderate)	Complicated nail cracks (nail cracks exposing underlying tissue), moderate overgrowth of nails or cuticles, moderate dry cuticles, infection or moderate injuries
3 (severe)	Underlying tissues exposed plus evidence of purulent discharge, deep pododermatitis, nail loss or severe injuries

#### Wound score

The wound score (WS) scale was developed by Schein et al. (2013) (Table 3). Each elephant was given a score of 0 (no wound), 1 (minor wounds), or 2 (major wounds). The number, location, types and causes of any open wounds were noted. Wounds were classified into six types: abrasion (the epidermis has been rubbed off); ulcer (a local excavation of the tissue surface that contains inflammatory exudate); abscess (a collection of pus enclosed in an area of inflamed tissue); laceration (irregular shaped wound with possible tissue loss); penetrating (a wound caused by a sharp, usually slender object that passes through the skin into the underlying tissues); and incision (a cut in the skin caused by sharp, cutting materials) (Scorer, 2014; Studdert, Gay & Blood, 2011).

**Table 3:** Scoring system for assigning wound scores based on Schein et al. (2013).

Score	Description
0	No lesions
1 (minor)	Minor wounds such as scrapes, scratches, superficial wounds or mild bleeding, some serous discharge
2 (major)	Major wounds such as severe bleeding, severe infection with pus, deep destruction of tissue, exposing muscle or bone

### Statistical analysis

Median and percentage data were calculated for body condition, foot health and skin WSs. Generalized estimating equations (GEE) were conducted for fitting marginal regression models using an R program (R Development Core Team, 2018), package multgee, function ordLORgee, which is appropriate for repeated multinomial variables with ordinal response categories (Touloumis, 2015) like the factors associated with physical health and welfare. Mean ± SD and range were used to describe the continuous variables. Management factors were selected as covariates in relation to Body condition score (BCS), Foot health score (FS) and WS (Table 4). Selection of variables for BCS and FS was adapted from Morfeld et al. (2016) and Miller, Hogan & Meehan (2016), respectively. Camps were treated as random effects. The selection process began by a univariate analysis of each variable. Any variable having a significant univariate test at *P* < 0.15 was selected as a candidate for the multivariate analysis, statistical significance for which was set at *P* < 0.05. Sex and Age were included in the multivariate model as confounders. Outputs from the GEE analysis were interpreted by using Odds Ratio (OR), which were calculated by the exponential value of the estimate.

**Table 4:** Explanatory variables for GEE models of body condition score (BCS), foot score (FS), and wound score (WS), and descriptive statistics of the continuous variables.

Variable name	Description	Mean ± SD	Range	GEE Models		
				BCS	FS	WS
Sex	Female or male			✓	✓	✓
Age	Age of elephant (years)	33.9 ± 11.2	5–65	✓	✓	✓
Work hour	Duration of work when elephants interacted with tourists per day (h)	5.3 ± 2.1	0–9	✓	✓	✓
Chain hour	Duration of chaining per day (h)	14.9 ± 7.3	0–23.5	✓	✓	
Walk distance day	Walking distance during working period per day (m)	4,215.0 ± 3,295.8	10–12,000	✓	✓	✓
Walk time day	Walking time during working period per day (min)	121.2 ± 86.3	10–450	✓	✓	✓
Roughage day	Amount of roughage per day (kg)	142.7 ± 55.8	12–500	✓		
Supplement day	Amount of supplement per day (kg)	21.1 ± 19.3	5–100	✓		
Free foraging	Ability to forage in the forest or grass field everyday			✓		
Feed day	Number of feedings of roughage during the day			✓		
Feed night	Number of feedings of roughage during the night			✓		
Feed total	Sum of feedings of roughage during the day and night			✓		
Water night	Ability to access water during the night; yes or no			✓		
Floor day	Type of floor in rest area during daytime; ground or concrete				✓	
Floor night	Type of floor in rest area during nighttime; ground, concrete, or sand				✓	✓
Floor work	Type of floor during working; ground only or mix between ground and concrete				✓	
Hill	Walking up or down hill during working or exercise; yes or no				✓	
Hook	Using a hook to control an elephant; yes or no					✓

**Note:**

Data from elephants during periods of musth (*n* = 9) and pregnancy (*n* = 2) were excluded.

Data for elephants during periods of musth and gestation were excluded from analysis because of alterations in nutrition and working management; that is, isolation of bulls, increased food provisioning and reduced work load for females. During the study period, nine bulls came into musth and two females conceived; only months associated with these states were excluded.

## Results

### Descriptive statistics of physical health and welfare parameters

**Figure 1:**
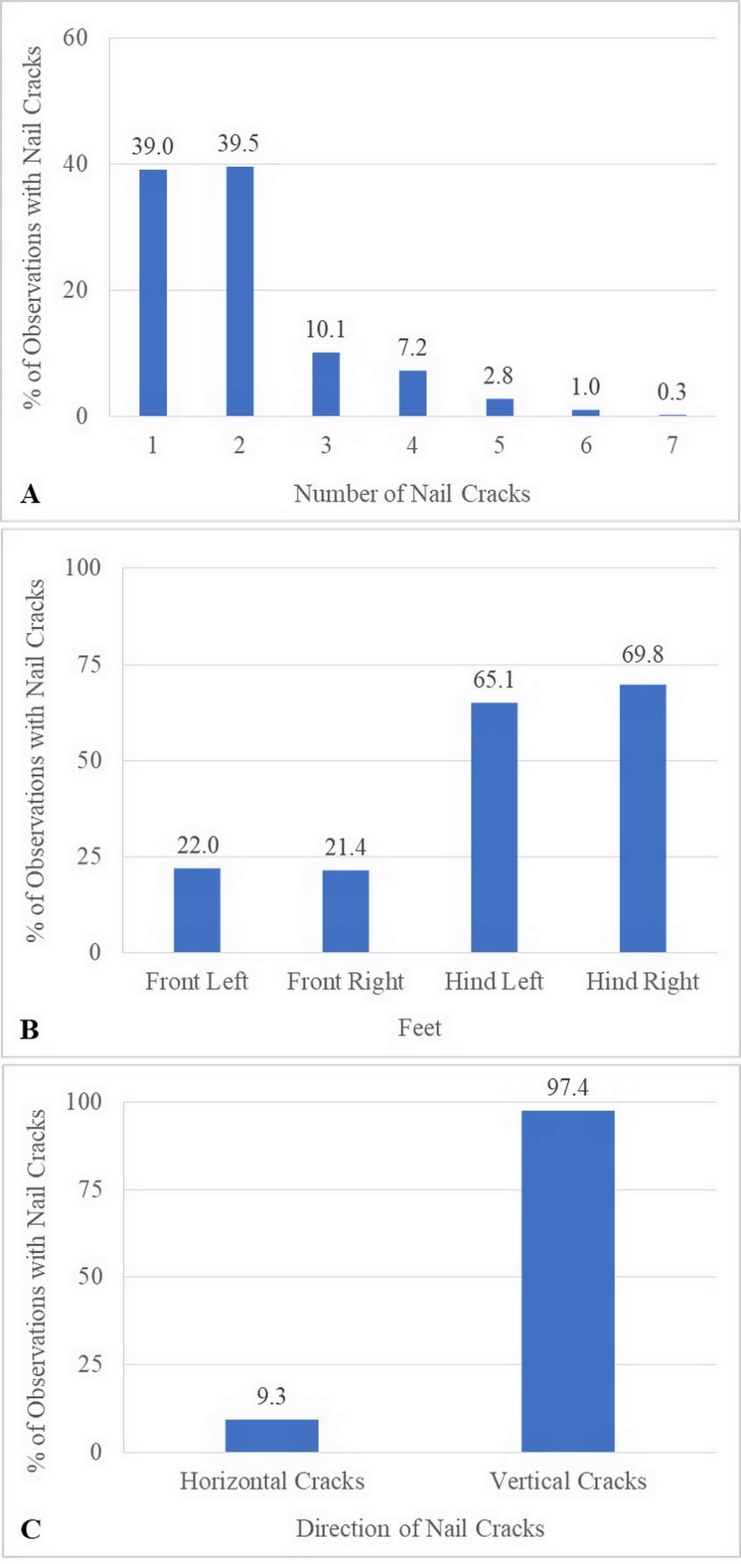
Characteristics of nail cracks of 387 observations from six times evaluation over a 15-month period. (A) Percentage of observations with nail cracks by the total number of cracks. (B) Percentage of observations with nail cracks by foot location. (C) Percentage of observations with nail cracks with vertical and horizontal cracks.

**Figure 2:**
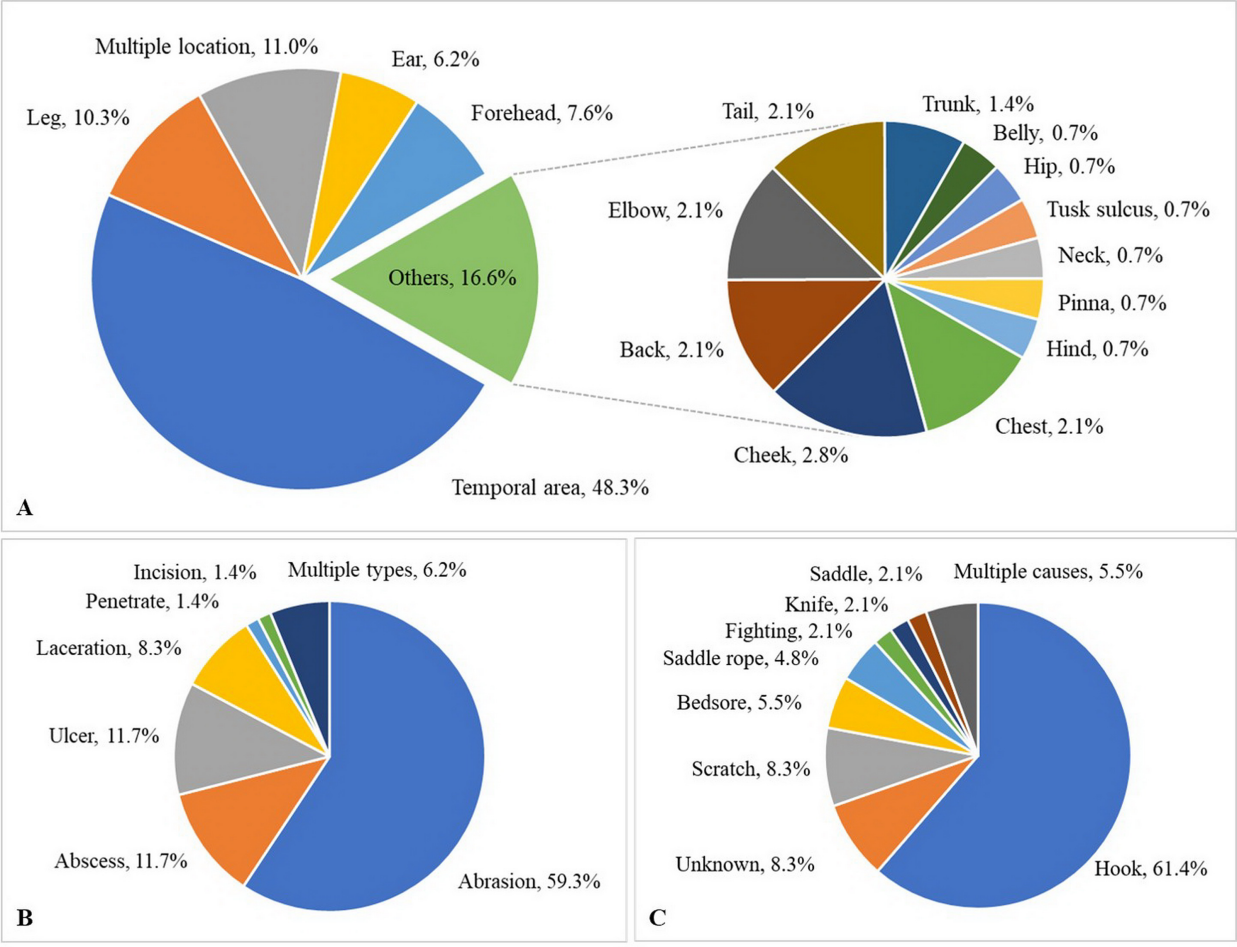
Characteristics of wounds of 387 observations from six times evaluation over a 15-month period (percentage). (A) Wound location, (B) Wound type and (C) Cause of wound.

**Figure 3:**
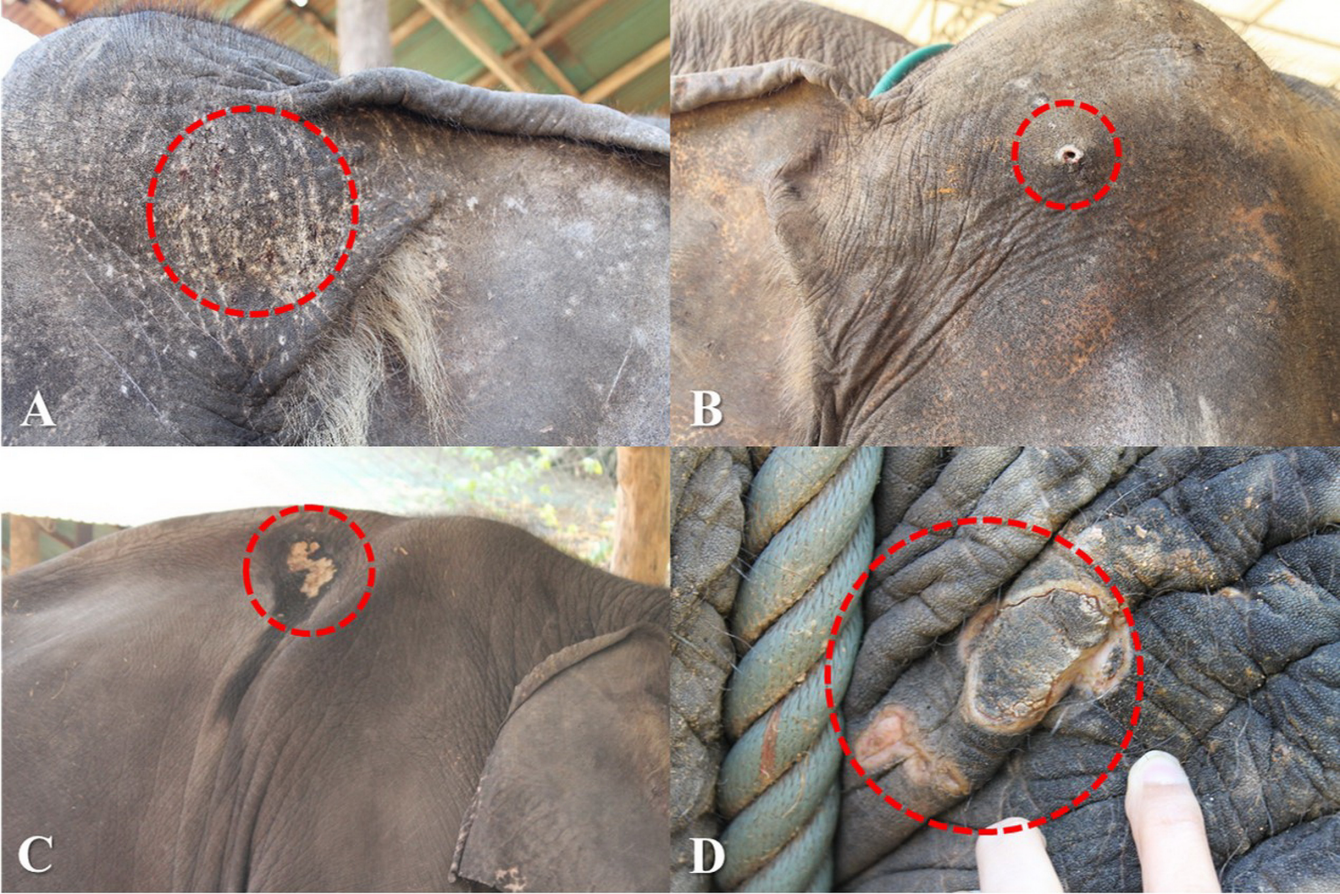
Skin lesions from inappropriate restraining method and improper use of equipment. (A) Abrasion wounds and (B) A laceration wound in the head region caused by hooks. (C) Ulcers in the back and (D) Breast region caused by saddle equipment. Photography by Pakkanut Bansiddhi.

Over the 1-year study, a total of 638 observations were obtained on 122 elephants. The mean number of observations per elephant was 5.2 ± 1.4 (range, 1–6). Median BCS was 4 (range 2–5). The majority of elephants were BCS >3, and none were BCS = 1 (Table 5). Across elephants, BCS increased as the study progressed from 18.9% at Time 1–46.5% at Time 6. Median and mean FS were both 1 (range 0–3: Table 5). In 61% of the observations (*n* = 106 elephants across 387 observations), nail cracks were observed. Of those, 79% (*n* = 304 observations) involved one to two nail cracks. Nail cracks were more common in the hind feet (left 65%; right 70% of the observations) than front feet (left 22%; right 21%) and developed in a vertical (97%) rather than horizontal direction (9%) (Fig. 1). Median WS was 0 (range 0–2), with the majority of elephants having no visible wounds. Wounds were noted in 23% of the observations (*n* = 65 elephants across 145 observations) (Table 5). The most common wounds were abrasions (59% of the observations), found mainly in temporal region next to forehead, and were most likely related to hook use (Fig. 2). A total of 73 elephants (60% of the elephants, *n* = 365 observations) were controlled by hooks. Of those, 27% of the observations (*n* = 53 elephants across 97 observations) involved wounds in the temporal area (74%, *n* = 72 observations), ears (11%, *n* = 10), forehead (9%, *n* = 9), legs (1%, *n* = 1), tail (1%, *n* = 1) and multiple areas (4%, *n* = 4). Types of hook wounds included abrasions (80%, *n* = 77 observations) (Fig. 3A), lacerations (12%, *n* =12) (Fig. 3B), ulcer (4%, *n* = 4), abscess (3%, *n* = 3) and multiple types (1%, *n* = 1). A total of 40 elephants (*n* = 200 observations) were used in a saddle riding program, and in only 5% of the observations (*n* = 6 elephants across nine observations) were wounds observed; these included ulcers on the back (56%, *n* = 5 observations) (Fig. 3C) or chest (44%, *n* = 4) (Fig. 3D), areas that were in contact with saddle pads or chest pieces.

**Table 5:** Percentage of elephant body condition score, foot score, and wound score categories from six times of scoring.

Score	Category	Time 1		Time 2		Time 3		Time 4		Time 5		Time 6		Total	
		*n* = 106	%	*n* = 108	%	*n* = 111	%	*n* = 105	%	*n* = 107	%	*n* = 101	%	*n* = 638	%
Body condition score	1	0	0.0	0	0.0	0	0.0	0	0.0	0	0.0	0	0.0	0	0.0
	2	2	1.9	5	4.6	9	8.1	6	5.7	6	5.6	4	4.0	32	5.0
	3	30	28.3	27	25.0	28	25.2	32	30.5	29	27.1	19	18.8	165	25.9
	4	54	50.9	53	49.1	48	43.2	34	32.4	36	33.6	31	30.7	256	40.1
	5	20	18.9	23	21.3	26	23.4	33	31.4	36	33.6	47	46.5	185	29.0
Foot score	0	34	32.1	42	38.9	39	35.1	50	47.6	32	29.9	33	32.7	230	36.1
	1	41	38.7	40	37.0	51	45.9	48	45.7	54	50.5	44	43.6	278	43.6
	2	29	27.4	22	20.4	19	17.1	6	5.7	20	18.7	21	20.8	117	18.3
	3	2	1.9	4	3.7	2	1.8	1	1.0	1	0.9	3	3.0	13	2.0
Wound score	0	70	66.0	78	72.2	89	80.2	87	82.9	86	80.4	83	82.2	493	77.3
	1	27	25.5	23	21.3	17	15.3	15	14.3	14	13.1	14	13.9	110	17.2
	2	9	8.5	7	6.5	5	4.5	3	2.9	7	6.5	4	4.0	35	5.5

**Note:**

Data from elephants during periods of musth (*n* = 9) and pregnancy (*n* = 2) were excluded.

### Factors associated with physical health and welfare parameters

Results of univariate and multivariate GEE analyses of variables associated with BCS (Table 6), FS (Table 7) and WS (Table 8) are presented. Variables affecting BCS in the univariate analysis were sex, chain hour, supplement day, free foraging, feed night, feed total and water night. In the final multivariate analysis, the combination of sex and water night were associated with higher BCS. Females were 4.394 times more likely to have a higher BCS compared to males (*P* < 0.001). Elephants that did not have the ability to access water during the night had an 87% decreased risk of having high BCS as compared to elephants that did have access water at night (OR = 0.130, *P* = 0.001) (Table 6). Variables associated with FS in the univariate analysis were work hour, walk distance day and floor work. Age was the only variable significant in the multivariate model. Increasing age was found to be associated with an increased odds of having a high FS (OR = 1.023, *P* = 0.031) (Table 7). Variables affecting WS in the univariate analysis were sex, age, hook and floor night. In the multivariate analysis, the combination of age, hook and floor night significantly affected WS. Increasing age was found to be associated with an increased odds of having a higher WS (OR = 1.040, *P* = 0.001). Elephants that were not controlled by hooks had a 70% decreased risk of having a higher WS as compared to elephants that were controlled by hooks (OR = 0.298, *P* < 0.001). Elephants that rested on a sand floor (*n* = 19 elephants) had an 85% decreased risk of having a high WS compared to elephants that rested on a compact dirt floor (OR = 0.146, *P* = 0.006) (Table 8).

**Table 6:** Univariate and multivariate GEE analyses of variables associated with body condition score.

Variable	*N*	Univariate analysis			Multivariate analysis		
		Estimate	Odds ratio	*P*-value	Estimate	Odds ratio	*P*-value
Sex							
Male	33	Reference					
Female	89	1.664	5.282	<0.001	1.480	4.394	<0.001
Age	122	−0.001	0.999	1.000	−0.019	0.981	0.163
Work hour	122	0.007	1.007	0.736			
Chain hour	122	−0.056	0.946	0.001	−0.008	0.992	0.706
Walk distance day	122	−0.001	0.999	0.615			
Walk time day	122	0.001	1.001	0.965			
Roughage day	122	0.003	1.003	0.243			
Supplement day	122	0.014	1.014	0.075	0.012	1.012	0.212
Free foraging							
Yes	43	Reference					
No	79	−0.702	0.496	0.020	−0.609	0.544	0.079
Feed day							
1	11	Reference					
2	39	0.064	1.066	0.876			
3	30	−0.252	0.777	0.537			
4	29	−0.571	0.565	0.193			
5	13	0.179	1.196	0.732			
Feed night							
Yes	86	Reference					
No	36	1.274	3.573	<0.001	0.211	1.235	0.618
Feed total							
2	25	Reference					
3	31	−0.728	0.483	0.102	−0.294	0.745	0.562
4	36	−0.889	0.411	0.037	−0.473	0.623	0.326
5	20	−1.125	0.325	0.037	−0.902	0.406	0.089
6	10	−0.804	0.448	0.137	0.305	1.356	0.584
Water night							
Yes	19	Reference					
No	103	−2.046	0.129	<0.001	−2.037	0.130	0.001

**Note:**

Data from elephants during periods of musth (*n* = 9) and pregnancy (*n* = 2) were excluded.

Variables having a *P*-value < 0.15 in the univariate analysis were included in the multivariate analysis.

**Table 7:** Univariate and multivariate GEE analyses of variables associated with foot score.

Variable	*N*	Univariate analysis			Multivariate analysis		
		Estimate	Odds ratio	*P*-value	Estimate	Odds ratio	*P*-value
Sex							
Male	33	Reference					
Female	89	−0.089	0.915	0.757	−0.141	0.868	0.615
Age	122	0.016	1.016	0.159	0.023	1.023	0.031
Work hour	122	0.045	1.046	0.093	0.031	1.032	0.372
Chain hour	122	−0.001	0.999	0.976			
Walk distance day	122	0.001	1.001	0.147	0.001	1.001	0.539
Walk time day	122	0.001	1.001	0.638			
Floor day							
Ground	66	Reference					
Concrete	56	−0.074	0.928	0.769			
Floor night							
Ground	50	Reference					
Concrete	53	0.183	1.201	0.515			
Sand	19	−0.344	0.709	0.338			
Floor work							
Ground	76	Reference					
Ground and concrete	46	0.477	1.612	0.070	0.507	1.661	0.066
Hill							
Yes	91	Reference					
No	31	−0.314	0.730	0.284			

**Note:**

Data from elephants during periods of musth (*n* = 9) and pregnancy (*n* = 2) were excluded.

Variables having a *P*-value < 0.15 at the univariate analysis were included in the multivariate analysis.

**Table 8:** Univariate and multivariate GEE analyses of variables associated with wound score.

Variable	*N*	Univariate analysis			Multivariate analysis		
		Estimate	Odds ratio	*P*-value	Estimate	Odds ratio	*P*-value
Sex							
Male	33	Reference					
Female	89	−0.824	0.439	0.005	−0.546	0.579	0.063
Age	122	0.023	1.023	0.048	0.039	1.040	0.001
Work hour	122	−0.062	0.940	0.164			
Walk distance day	122	−0.001	0.999	0.536			
Walk time day	122	0.001	1.001	0.428			
Hook							
Yes	73	Reference					
No	49	−1.778	0.169	<0.001	−1.212	0.298	<0.001
Floor night							
Ground	50	Reference					
Concrete	53	0.398	1.489	0.154	0.212	1.236	0.423
Sand	19	−2.567	0.077	<0.001	−1.926	0.146	0.006

**Note:**

Data from elephants during periods of musth (*n* = 9) and pregnancy (*n* = 2) were excluded.

Variables having a *P*-value < 0.15 at the univariate analysis were included in the multivariate analysis.

## Discussion

This is the first study to examine the effect of management practices on the health and welfare of tourist elephants by using an epidemiological approach. Study subjects were followed over time with repeated monitoring of risk factors and outcomes; that is, BCS, FS and WS, which made observing changes more robust.

When using a 5-point scale, the “ideal/normal” BCS = 3; BCS = 1–2 equates to “underweight/thin” and “overweight/obese” includes BCS = 4–5 (Morfeld et al., 2016). BCS of elephants in this study was generally high with a median BCS = 4, which was comparable to elephants in North American zoos (Morfeld et al., 2016), but higher than free-ranging elephants in India (Pokharel, Seshagiri & Sukumar, 2017) that used the same scoring system. However, proportionally, Thailand elephants were in better body condition compared to zoo elephants, with 69% at a BCS of four or five compared to 74% in North American zoos. Captive elephants, on average, have higher body condition than free ranging elephants, presumably because of more consistent, high quality diets, but also fewer exercise opportunities. For that reason, comparatively, the majority of western zoo elephants are overweight or obese (Harris, Sherwin & Harris, 2008; Morfeld et al., 2016). While the proportion of FSs and WSs were stable throughout the study period, the percentage of elephants with a BCS of five more than doubled, from 18.9% at Time 1–46.5% at Time 6. One reason for this may be that work duration, walking distance and walking time decreased as the study progressed, while the amount of roughage and supplements remained constant. The decrease in working intensity may have been related to lower tourist numbers in those months of the study. Although those data were not available, in October 2016, TripAdvisor and its booking service, Viator, announced they will no longer sell tickets to hundreds of attractions where travelers come into contact with wild animals or endangered species held in captivity. This announcement could have led to a decrease in tourist numbers during Time 4–6 (October 2016 – May 2017), which then resulted in higher observed BCSs. If so, this effect deserves further investigation, and management adjustments may be needed to account for it.

In our study, females had higher BCS than males, which is similar to Asian elephants in North American zoos (Morfeld et al., 2016). Fat deposits enable a female mammal to bear the energy costs of gestation and lactation, which is important to reproductive success (Heldstab, Van Schaik & Isler, 2017). In addition, male elephants lose weight during musth because of decreased foraging and active interest in females in relation to elevated androgens, and to subsequent catabolism of triglycerides (Goodwin et al., 2015). From our interview, mahouts reported keeping body condition of males lower in an effort to control musth symptoms and reduce aggressive behavior.

Having a water source at night was a significant factor related to higher body condition in elephants. Although one management goal is to reduce the risk of a high BCS because of associated health concerns (Morfeld & Brown, 2016; Morfeld & Brown, 2017; Morfeld et al., 2016), water is important for maintaining adequate body condition in underweight elephants. We were unable to correlate amounts of water consumed per day to BCS, but this could be important for proper welfare management. One of the “Five Freedoms” for animals is the freedom from hunger and thirst by providing adequate and ready access to fresh water (Farm Animal Welfare Council, 1979). Dunkin et al. (2013) found that cutaneous evaporative water loss increased with increasing air temperature across the body of elephants and they used additional water sources to increase evaporative cooling. Because of the high physical activity experienced by some elephants in the tourist industry and the hot, tropical climate in Thailand, providing water both day and night might help to maintain full health and vigor. We were not expecting a relationship between what we consider to be an unhealthy BCS and access to water at night. Further evaluation of the data suggests it might be related to other management practices, and not a direct cause and effect. Specifically, we found elephants that had access to water at night also received greater amounts of roughage and high-calorie supplements during the day, worked fewer hours and exercised less than those that did not have water at night. So, more food and a lower work intensity is more likely the cause of higher BCSs rather than having access to water at night. A surprising finding from the management survey (Bansiddhi et al., 2018) was that only 18% of camps had a water source for elephants at night, so understanding how this limitation affects long-term health and welfare is warranted.

The amount of supplement offered to elephants was associated with higher BCS in the univariate analysis. The most common supplements for elephants in this population were bananas and sugarcane (Bansiddhi et al., 2018), which possess high concentrations of sucrose and other soluble sugars that could contribute to weight problems (Norkaew et al., 2018). Limiting the amount of high-calorie treats and using lower calorie supplements, such as tamarind, watermelon, pumpkin, pineapple and cucumber, which are easy to obtain locally (Bansiddhi et al., 2018; Phuangkum, Lair & Angkawanith, 2005), is recommended if tourists must feed elephants.

Elephants that had a chance to forage regularly had an increased risk for high BCS in the univariate analysis. For underweight elephants, this can help improve their body condition. Having foraging opportunities is important to elephants as it can increase the variety of foods and herbs they eat and is supportive of natural behavior. Foraging enrichment is considered one of the most effective strategies to improve welfare and reduce stereotypies and other abnormal repetitive behaviors in captive animals (Van Zeeland et al., 2013), including elephants (Morfeld et al., 2016). Thus, to maintain good body condition, elephant camps should promote foraging and not feed elephants a lot of supplementary treats. An increase in the number of feedings per day and feeding elephants at night, however, were associated with lower BCS in the univariate analysis. In animal experiments, Anderson et al. (1980) found significantly less body weight gain with higher eating frequency in rat pups. In humans, it is a common practice for clinicians to recommend increasing meal frequency as a strategy for weight management and to improve metabolic parameters. Frequent meals have been further proposed to reduce the occurrence of excess caloric consumption and provide better glucose control and reduced insulin secretion (Kulovitz et al., 2014). Another important factor associated with BCS in zoo elephants is feeding schedules; an unpredictable schedule was associated with a 69% decrease risk of BCS four or five as compared to elephants with a predictable feeding schedule (Morfeld et al., 2016). For tourist elephants, most were fed roughage by their mahouts on a fairly predictable feeding schedule, although feeding of supplementary treats by tourists was on an unpredictable schedule. Thus, it is unclear if a similar relationship between BCS and unpredictable feeding schedules applies to tourist elephants.

One unexpected finding in the univariate analysis was that elephants not constrained by prolonged chaining had an increased risk for high BCS. One consequence of chaining in elephants is the development of stereotypic behaviors (Friend & Parker, 1999; Gruber et al., 2000; Schmid, 1995; Varadharajan, Krishnamoorthy & Nagarajan, 2016), which consist of repetitive movements that may burn more calories. Data on this population showed that elephants that presented stereotypic behaviors had lower BCS than elephants that did not (GEE, *n* = 122, *P* = 0.02).

In examinations of foot health, the majority of observations (44%) found mild problems with uncomplicated nail cracks or injuries; in only 2% of the observations were severe foot problems observed. These results were comparable to a study by Todd (2015) that scored 74 elephants in three camps in Chiang Mai by the same system and found the majority (64%) had mild and 3% had severe foot problems. In our study, more than half of observations (61%) had nail cracks. This finding was similar to a survey of tourist elephants in India that found nail cracks in 61% (Sasmal, 2018). Foot problems do occur in wild elephants, but they are not common (Fowler, 2001). Benz (2005) noted that captive elephants have a thinner sole and pad horn layer in the weight-bearing surface in comparison to wild elephants, which encourages foot disorders in captive elephants and is caused by a floor in zoos that is too hard and abrasive. That study also evaluated wild elephants and found no evidence of cracks, fissures, holes or other pathological alterations. However, micro-cracks were apparent in all animals, both captive and wild, suggesting they result from normal wear. According to assertions by various vets working in the wild in Sri Lanka and South Africa, wild elephants’ foot problems are usually related to trauma (penetration by shot, traps or sharp objects on the ground, but also burn wounds) and resulting infections (Benz, 2005).

We did not find the forelimbs to be more affected by nail cracks than the hind limbs, despite bearing a greater proportion (about 61%) of body weight in a standing position (Genin et al., 2010) or higher in mean peak pressure magnitudes (by about 5%) when walking on flat concrete (Panagiotopoulou et al., 2012). In fact, the opposite was true, and a majority of cracks were in the hind feet. However, during quadrupedal walking in most animals, the hind limbs become almost entirely responsible for providing propulsion to push themselves forward while the front limbs provide more braking (Granatosky et al., 2018; Tefera, 2012). The pressure from propulsion might induce nail cracks in the hind limbs, especially when elephants walk up hill. Similar to our findings, West (2001) found that nail cracks are common in hind feet in circus and zoo elephants. We found vertical cracks more often than horizontal cracks, which are generally caused by nail overgrowth, digging, kicking, or obesity (Fowler, 1993; Rutkowski, Marion & Hopper, 2001). Horizontal cracks, while more unusual, were found in 9% of elephants with nail cracks in this study. In horses, horizontal cracks are usually the result of an injury and rarely spread like vertical cracks (Thomas, 2006).

Increasing age was found to be associated with higher FS in the final model. Older elephants frequently develop foot problems, likely due to diseases like arthritis, and to reduced activity levels (West, 2001). Elderly captive elephants are predisposed to pododermatitis, an infectious process of the foot that takes 5–8 weeks longer to recover from in older elephants (Silva & Dangolla, 2006). In humans, age-associated nail changes and disorders are common in elderly patients. There is usually a tendency of the normally smooth nail plate texture to become progressively more friable with increasing age, resulting in fissuring, splitting and longitudinal superficial or deep striations (Cohen & Scher, 1992; Singh, Haneef & Uday, 2005).

In the univariate analysis, work hours and walking distance were associated with an increased risk of a high FS, possibly because an elephant’s foot and nail supports more weight and pressure when active than when standing still (Fowler, 2001). However, regular physical exercise, such as walking, does benefit captive elephants as it promotes muscle tone, flexibility, agility, stamina and a healthy weight, and it provides enrichment; lack of exercise is one of the alleged causes of foot problems (Fowler, 2008; Olson, 2004). Walking distances have been reported to range from 3.2 to 8.9 km per day in wild Asian elephants (Rowell, 2014) and 5.3 km per day in North American zoo elephants (Holdgate et al., 2016). Although the mean walking distance of 4.2 km per day in this study was comparable, there were elephants that walked up to 12 km per day. Those elephants that walked more than four km per day had a higher percentage of moderate to severe foot problems (FS of two and three) than elephants that walked less than four km per day (18%). However, Holdgate et al. (2016) and Miller, Hogan & Meehan (2016) found no correlations between walking distance and foot health in elephants in North American zoos. The authors noted that those data were not collected at the same time, whereas they were in this study. So, it is possible that measures of foot health taken coincident with walking distance measurements more accurately reveal these associations. It must be noted that walking distance and walking time in this study were estimated by mahouts and only during the working period, not during free-foraging or when taken for bathing and drinking during rest periods.

Having concrete floors in walking routes was associated with higher FS in the univariate analysis. Similarly, a study in zoo elephants demonstrated a significant relationship between time spent on hard substrate and foot problems (Miller, Hogan & Meehan, 2016). In farm animals (e.g., cattle and pigs), excessive walking on concrete causes foot and claw disorders, stretches in the white line (lamina horn that connects the sole to the hoof wall) and wearing down of the sole, thus weakening the junction between the wall and sole of the foot (Mills & Marchant-Forde, 2010; Ribo & Serratosa, 2009).

Most wounds were found in the head region; that is, the forehead and temporal area next to the forehead, and the ears, all places where equipment (e.g., hooks) is used. We also found penetrating and incision wounds caused by knives, the other tool commonly carried by mahouts. It was not possible to determine misuse of hooks in sensitive areas of the elephant’s skin (less than one cm thick), including inside the ears or mouth, behind the ears, in and around anus, under the chin and around the feet (Doyle, 2014; Shoshani, 1982), but it is possible they may be present. Using hooks where skin is thicker (e.g., around the forehead), may require more force that can lead to pronounced injuries. It must be noted that in 73% of the observations where mahouts carried a hook, the elephants had no associated wounds. Thus, misuse of hooks may not be as widespread as animal protection groups suggest. Still, our findings indicate there is overuse of equipment to control elephants in some camps or with some elephants. As such, using hooks was found to be associated with higher WSs. Therefore, it is imperative that mahouts be trained in proper use of the hook and how to control elephants without resorting to painful punishment. The knife should never be used to control an elephant, except when human life is in danger. The primary purpose of the bush knife is to cut food for the elephant, clear pathways, and cut firewood for the mahout (Phuangkum, Lair & Angkawanith, 2005), not to control elephants.

Skin lesions related to contact with saddle-related equipment were found in only 5% of observations at camps with a saddle program, which indicates that saddle use caused wounds in only a few elephants. This is much lower than a prevalence of 64% reported by Magda et al. (2015), which conducted a survey on 194 elephants from 18 tourism camps across Thailand. Their results showed that the use of a rice sack as padding was a significant risk factor for having an active lesion. It may be that camps have heeded recommendations from that study and made the changes necessary to avoid saddle injuries. Beside using gunnysacks, camps in northern Thailand generally use hammered bark, blankets, or sponge material as saddle padding (Bansiddhi et al., 2018), which might reduce the incidence of injuries. Shape of the backbone can also be a factor, with higher ridgelines being more susceptible to saddle injuries if saddles are not well designed, although this has yet to be studied in detail.

Increasing age had an expected association with higher WS, as Magda et al. (2015) also found that older elephants were more likely to have active lesions in association with saddle riding. In humans, many of the protective functions of skin decrease with age. Functional changes in aging skin include altered permeability, diminished sebum production, decreased inflammatory and immunological responsiveness, attenuated thermoregulation and reduced elasticity. These changes affect the rate and quality of healing (Gordon, 2014). Bedsores are one of the most common wounds in the elderly and form where the weight of the person’s body presses the skin against the firm surface of the bed (Kandha Vadivu, 2015). Because of their large body weight, lying down on hard floors might be a risk factor for developing sores in elephants. This was confirmed by our result that resting on sand floors at night was associated with low WS.

In the univariate analysis, males had a higher WS than females (24% of observations vs. 11%, respectively), most of these related to hook injuries. Males generally are more aggressive and need more intensive control. In addition, fights between males over territory or in competition for mates can cause serious wounds; that is, puncture wounds from tusks. We found one female and three males that had laceration, abrasion, or penetrating wounds from fighting.

## Conclusions

This is the first study linking tourist camp management practices with specific health and welfare outcomes in Asian elephants. Findings emphasize the need for some elephant camps to adjust management activities that affect body condition, foot health and wounding. It is important to strike a balance between work intensity, exercise opportunities and nutrition to prevent problems associated with obesity. Foot problems could be reduced by limiting walking on hard surfaces and establishing a regular foot care program. Carrying a hook is often necessary for the safety of elephants, mahouts and tourists in free-contact situations. However, protocols need to be enforced that prevent the misuse of equipment and unnecessary wounding. Furthermore, mahouts must be trained to use hooks properly and in a way that is not punitive. Saddles must fit properly to take pressure off the spine and with appropriate padding to prevent abrasions, and occasionally removed during the day to provide rest and relief of any pressure points.

Space and budget limitations may make it difficult for some camps to comply with all of these recommendations, but they must be more proactive to ensure these intelligent animals are managed in a way that meets physical and psychological needs. Simple acts, like changing from concrete to compact dirt or sand floors, especially for old elephants, and managing tourist interactions can make a big difference in the life of an elephant, without being financially onerous to owners. The results of this study will now be used to develop science-based welfare guidelines and elephant camp standards to aid in the management of elephants used in tourism. Future studies will use the survey data to further investigate associations between management factors and other behavioral and physiological welfare indicators to further refine these recommendations. Finally, we will work with elephant camps to help them improve welfare standards by providing education and training opportunities for owners and mahouts.

## Supplemental Information

10.7717/peerj.6756/supp-1 Supplemental Information 1 Questionnaire sheet used to record information during camp visits (S1). Questionnaire interviews with camp owners, managers and/or camp veterinarians were performed by using this questionnaire sheet to record information about camp activities, location, programs for tourists, numbers of elephants and elephant management.

10.7717/peerj.6756/supp-2 Supplemental Information 2 Questionnaire sheet used to record information from mahouts (S2). Questionnaire interviews with mahouts gathered information on management of their specific elephants; that is, work routine, restraint, rest area, feeding, watering and health care by using this questionnaire sheet.

10.7717/peerj.6756/supp-3 Supplemental Information 3 Raw data from the interviews and the examination applied for data analyses and preparation for Tables 1, 4, 5, 6, 7 and 8.

## Additional Information and Declarations

### Data Availability

The following information was supplied regarding data availability: The raw measurements are available in the Supplemental File.
