# Supplementary material for: Management factors affecting physical health and welfare of tourist camp elephants in Thailand
Source: PeerJ. 2019 Apr 25;7:e6756. doi: 10.7717/peerj.6756 (PMC6487189; doi:10.7717/peerj.6756)
Supplement: Supplemental Information 2 — Questionnaire interviews with mahouts gathered information on management of their specific elephants; that is, work routine, restraint, rest area, feeding, watering and health care by using this questionnaire sheet. [file peerj-07-6756-s002.docx]

**Supplement 2**

Questionnaire Sheet Used to Record Information from Mahouts

Name of interviewer .....................................................................

Name of interviewee ………………………………………

Date……………...............

**1. General information of the mahout**

1.1 Name................................................................

1.2 Sex: □ Male □ Female

1.3 Age...........................years old

1.4 Race............................................

1.5 Nationality............................................

1.6 Present address..................................................................................................

□ Mahout own □ Camp own

1.7 Native address....................................................................................................

1.8 How long have you been in Thailand?.........................................

1.9 Status: □ Single □ Married □ Have child........... persons

1.10 Level of education...................................................

1.11 Language proficiency:

□ Thai: □ Speaking □ Reading □ Writing

□ English: □ Speaking □ Reading □ Writing

□ Others................ □ Speaking □ Reading □ Writing

1.12 Work permit: □ No □ Yes

**2. Health information of the mahout**

2.1 Present illness/disease........................................................................................

2.2 Health check: □ Never □ Ever: When?............................................................

2.3 Tuberculosis test : □ Never □ Ever: When?....................................................

2.4 Drink alcohol: □ No □ Yes: When?.................................................................

2.5 Smoke cigarette: □ No □ Yes: When?............................................................

2.6 Other drugs: □ No □ Yes: What kind?.............................................................

**3. Job experience**

3.1 How long have you worked in this camp?........................................................

3.2 How could you work in this camp?...................................................................

3.3 How long have you been a mahout?..................................................................

3.4 Why you work as a mahout?.............................................................................

3.5 How could you come to be a mahout?..............................................................

3.6 How many elephants have you taken care?.................. elephants

3.7 Have you ever hurt from this job?: □ No

□ Yes: When?..............................................................................................

How?................................................................................................

3.8 Have you ever joined any mahout training courses?: □ No

□ Yes: When?.............................................................................................. Where?.............................................................................................

3.9 Day off: □ No □ Yes: When?...........................................................................

**4. Economic status**

4.1 Main job.....................................................................

Income: □ Daily............................................. baht per day

□ Monthly........................................ baht per month

□ At time.......................................... baht per time

4.2 Part-time: □ No □ Yes: What?.........................................................................

Income: □ Daily............................................. baht per day

□ Monthly........................................ baht per month

□ At time.......................................... baht per time

4.3 Total income per month.......................................baht

4.4 Do you think your pay is fair?: □ No □ Yes

Why?............................................................................................................

4.5 Highest income: □ per day................................ baht

□ per month............................ baht

4.6 Lowest income: □ per day................................ baht

□ per month............................ baht

4.7 Facility:

□ Car □ Motorcycle □ TV □ Refrigerator

□ Mobile phone □ Internet □ Others.....................................

4.8 Debt status: □ No debt

□ Creditor................... baht

□ Debtor..................... baht: Why?..............................................................

4.9 Loaning money: □ No

□ Yes: Source: □ Lender □ Government □ Others....................................

**5. Information of this elephant**

5.1 How many elephants that you have to take care?.................... elephants

5.2 How long have you taken care this elephant?...................................

5.3 Elephant name: □ Plai □ Sridor □ Pung.............................................

5.4 Sex: □ Male □ Female

5.5 Age.........................................

5.6 Where this elephant come from?.......................................................................

5.7 Owner: □ Camp □ Renter

□ Mahout: □ Heritage □ Purchase: When?................. Price.....................baht

5.8 Do this elephant's family live in the same camp?: □ No

□ Yes: Which one?......................................................................................

5.9 Breeding history................................................................................................

5.10 Pregnant and calving history…......................................................................

5.11 History of disease and disorder………...........................................................

**6. Working of this elephant**

6.1 Do this elephant have to work?: □ No

□ Yes: □ Show

□ Trek

□ Riding: □ without saddle □ with saddle

□ Others...........................................................................................

6.2 (For non-working elephant)

Exercise: □ No

□ Yes: When?..............................................................................................

Distance/Area..................................................................................

Floor type: □ Ground □ Concrete □ Others…………………………

Slope: □ No □ Yes

6.3 (For working elephant)

Duration: High season: Start time………….. Stop time…………….

Low season: Start time………….. Stop time…………….

Breaking period: □ No

□ Yes: When?..................................................................................

Duration...............................................................................

Why?....................................................................................

(Show elephant)

Activity............................................................................................

How many shows per day?........................rounds

How long for each show?..............................................

(Walking elephant)

Ride by mahout: □ No □ Yes

How many rounds per day?........................rounds

How far for each round?.....................................

How long for each round?................................................

Floor type: □ Ground □ Concrete □ Others……………………

Slope: □ No □ Yes

(Riding elephant without saddle)

How many tourists?.......................persons

Riding position: □ Neck □ Back □ Both

Holding equipment: □ No □ Chest belt: Type...............................

(Riding elephant with saddle)

How many tourists?.......................persons

Saddle: □ Wood □ Steel □ Others.............

Saddle weight……………kg

Padding: □ Rice sack □ Bark of tree □ Sponge)

□ Plastic □ Others..............

Padding weight……………kg

Belt: □ Chest □ Tail □ Neck □ Others.......................................

Do you move the saddle out during break?: □ No

□ Yes: Duration................................

6.4 Special management for this elephant: □ No

□ Yes: What?............................................................................

6.5 Other work except from daily work: □ No

□ Yes: What?............................................................................

6.6 Injury from working: □ No

□ Yes: What?............................................................................

6.7 Day off: □ No

□ Yes: When?............................................................................

**7. Control and restraint**

7.1 Day time

Where is this elephant?..........................................................

Interaction with other elephants: □ No

□ Yes: How?....................................................................................

Chain:

□ No chained

Cage size/walking area.............................................

Floor type: □ Ground □ Concrete □ Others……………

Restraint: □ No

□ Yes: How?............................................................

□ Chained

When?: □ All time □ Break □ Others………………

Where?.................................................................................

Floor type: □ Ground □ Concrete □ Others……………

Chain material....................................

Length..................................meters

7.2 Night time

Where is this elephant?..........................................................

Interaction with other elephants: □ No

□ Yes: How?....................................................................................

Chain:

□ No chained

Cage size/walking area.............................................

Floor type: □ Ground □ Concrete □ Others……………

Restraint: □ No

□ Yes: How?............................................................

□ Chained

When?: □ All time □ Break □ Others………………

Where?.................................................................................

Floor type: □ Ground □ Concrete □ Others……………

Chain material....................................

Length..................................meters

7.3 Can you enter to control this elephant:

□ No: Why?.................................................................................................

□ Yes: Restraint equipment: □ No □ Hook □ Nail □ Knife

□ Sling shot □ Chain □ Others…………...

If use equipment, When?:

□ During work □ During Approach □ Others……………

Injury from restraint: □ No

□ Yes: What?...................................................................................

**8. Feeding**

| Type of food | Feeder: mahout/tourist | Amount per day | Frequency | Amount per meal |
| --- | --- | --- | --- | --- |
|  |  |  |  |  |
|  |  |  |  |  |
|  |  |  |  |  |
|  |  |  |  |  |
|  |  |  |  |  |

**9. Drinking**

9.1 How many time per day that this elephant can drink water?.........time per day

□ not fix

9.2 How can this elephant drink water? □ by themselves □ by mahout

9.3 Source of water: □ river □ pond □ underground water

□ tap water □ Others............................................

9.4 Can this elephant drink water by themselves?

Day time: □ No □ Yes

Night time: □ No □ Yes

**10. Routine care**

10.1 Do you clean this elephant?: □ No

□ Yes: By whom?.............................................................................

How?.....................................................................................

How often?............................................................................

10.2 Health check: □ No

□ Yes: By whom?.............................................................................

How?.....................................................................................

How often?............................................................................

10.3 Foot care: □ No

□ Yes: By whom?.............................................................................

How?.....................................................................................

How often?............................................................................

10.4 Do you clean the rest area?: □ No

□ Yes: By whom?.............................................................................

How?.....................................................................................

How often?............................................................................

10.5 Do you play with this elephant?: □ No

□ Yes: How?.....................................................................................

How often?............................................................................

10.6 What you do if something wrong with your elephant?:

□ Treat by mahout □ Call for vet □ Call for staff of clinic

**11. Elephant behavior**

11.1 Habit.....................................................................

11.2 Do your elephant have time to play?: □ No

□ Yes: What?: □ River □ Mud □ Other elephants □ Others……………

How often?........................................................................

11.3 Do you think your elephant want to be around with other elephants?: □ No

□ Yes: How?.................................................................................

11.4 Aggressive behavior: □ No

□ Yes: When?................................................................................

11.5 Stereotypes: □ No

□ Yes: What kind?....................................................................

When?...........................................................................

When did the behavior start?........................................

**12.** **Attitude about elephants and your job**

12.1 Do you love elephants?....................................................................................

12.2 Do you think elephants are clever?..................................................................

12.3 Where should elephants live?..........................................................................

12.4 Should elephants work? What type?...............................................................

12.5 Should tourist able to touch elephants?...........................................................

12.6 Is your elephant in well-living?.......................................................................

12.7 What about restraint equipme..........................................................................

12.8 When we should stop working elephants? At what age?................................

12.9 What about mahout job?..................................................................................

12.10 Do you think you are a good mahout?...........................................................

12.11 How income and welfare of mahouts should be?..........................................

12.12 Are you and your family in well-living?.......................................................

12.13 What about your future?................................................................................
